# Sudden hypoxemia after uneventful laparoscopic cholecystectomy: another form of SAM presentation

**DOI:** 10.1186/s12871-015-0031-y

**Published:** 2015-04-16

**Authors:** Yoshihisa Fujita, Nobuyuki Kagiyama, Yuka Sakuta, Masatsugu Tsuge

**Affiliations:** 1 Department of Anesthesiology & ICM, Kawasaki Medical School, Okayama, Japan; 2 Division of Cardiology, Internal Medicine, Kawasaki Medical School, Department of Cardiology, Present affiliation: The Sakakibara Heart Institute of Okayama, Okayama, Japan

**Keywords:** Pulmonary edema, Hypoxia, Systolic anterior motion, Echocardiography, MRI

## Abstract

**Background:**

Perioperative dynamic left ventricular outflow obstruction associated with systolic anterior motion of the mitral valve is well recognized as a cause for unexplained sudden hypotension in perioperative settings, even without underlying heart diseases such as hypertrophic obstructive cardiomyopathy. We treated a patient who experienced sudden hypoxemia without severe hypotension during emergence from anesthesia after an uneventful laparoscopic cholecystectomy.

**Case presentation:**

A 65-year-old female patient with a history of hypertension presented a sudden decrease in oxygen saturation to 80% after an uneventful cholecystectomy. Although a portable chest radiograph showed bilateral hilar pulmonary infiltrates consistent with pulmonary edema, we explored the underlying cause, i.e., systolic anterior motion of the mitral valve and left ventricular outflow tract obstruction with bedside transthoracic echocardiography. We speculate that dynamic mitral regurgitation resulted in pulmonary edema and, thereby, hypoxemia in this case without severe hypotension.

**Conclusions:**

Careful bedside examination with transthoracic echocardiography was useful in making diagnosis and in guiding appropriate therapy for this patient. Clinicians should be aware that systolic anterior motion of the mitral valve may present as unexplained sudden hypoxemia in the perioperative setting.

**Electronic supplementary material:**

The online version of this article (doi:10.1186/s12871-015-0031-y) contains supplementary material, which is available to authorized users.

## Background

Perioperative dynamic left ventricular outflow obstruction (LVOTO) associated with systolic anterior motion (SAM) of the mitral valve is well recognized as a cause for unexplained sudden hypotension in perioperative settings, even without underlying heart disease such as hypertrophic cardiomyopathy (HOCM) or apical ballooning syndrome [1-4]. We treated a woman who experienced a sudden decrease in oxygen saturation (SpO_2_) to 80% without severe hypotension during emergence from anesthesia after an uneventful laparoscopic cholecystectomy. SAM-related mitral regurgitation (MR) was suspected to be responsible. Our case demonstrates that SAM may present as unexplained hypoxemia in the perioperative setting.

## Case presentation

A 65-year-old woman with a 5-year history of hypertension and depression was scheduled for laparoscopic cholecystectomy under general anesthesia. Anesthesia was induced with propofol, and the airway was secured with a size #3 ProSeal laryngeal mask airway (PLMA). Anesthesia was maintained with desflurane at 4% in an oxygen–air mixture (FiO_2_ = 50%), and analgesia was achieved by continuous infusion of remifentanil at a rate of 0.16-0.24 μg · kg^-1^ · min^-1^ supplemented with fentanyl to a total of 0.4 mg. Muscle relaxation was attained with intermittent rocuronium. Oxygen saturation (SpO_2_) was kept at 99–100%, and blood pressure was relatively stable but fluctuated between 140/40 and 90/40 mmHg. The patient’s heart rate (HR) was 65–80 beats per minute during anesthesia. Surgery took 122 minutes, without measurable blood loss. A total of 1475 ml of crystalloid solution was infused intraoperatively. The anesthetic course was uneventful until the end of surgery.

Several minutes after the end of surgery and having confirmed spontaneous respiration, 200 mg of sugammadex was administered intravenously to reverse the neuromuscular blockade. Although spontaneous respiration resumed under 100% oxygen, SpO_2_ decreased to 80%, with an increase in arterial pressure to 180/100 mmHg. The attending anesthesiologist, who suspected that a poorly fitted PLMA was the cause of hypoxemia, replaced the size #3 PLMA with a size #4 PLMA, and there was a temporary increase in SpO_2_ to 92%. The PLMA was removed, and the patient was transferred to the recovery room with oxygen at 6 L/min via face mask.

**Figure 1:**
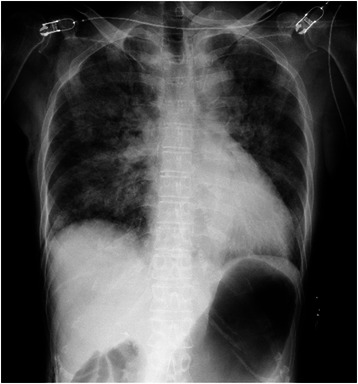
Portable chest radiograph. There were bilateral hilar pulmonary infiltrates, but the periphery of the lungs was relatively spared. Cardiac silhouette was not enlarged. There was air in the stomach.

**Figure 2:**
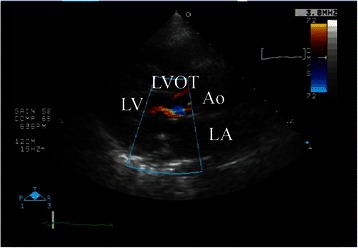
Bedside transthoracic echocardiography with color-flow Doppler mapping in the post-anesthesia care unit (left parasternal long axis view). Slight mosaic flow signals in the left ventricular outflow tract was noted on colorflow Doppler mapping. LA = left atrium; LV = left ventricle; LVOT; left ventricular outflow tract; Ao = aorta.

**Figure 3:**
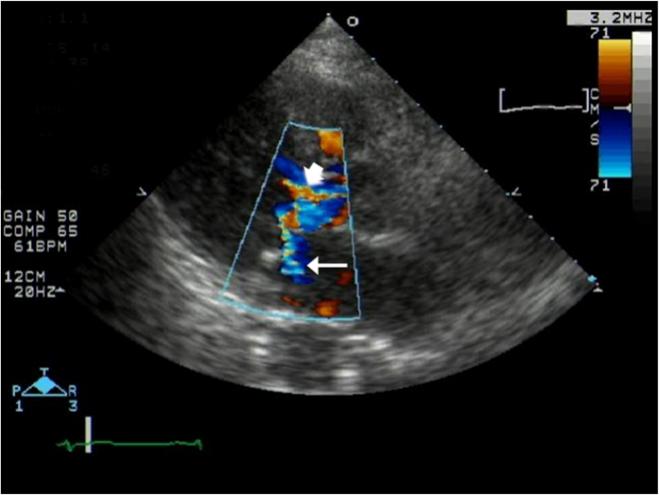
Bedside transthoracic echocardiography with color-flow Doppler mapping in the post-anesthesia care unit after sublingular nitrate (left parasternal long axis view). Significant mitral regurgitation jet in the posterior direction (arrow)and mosaic pattern left ventricular outflow (arrow head) appeared.

**Figure 4:**
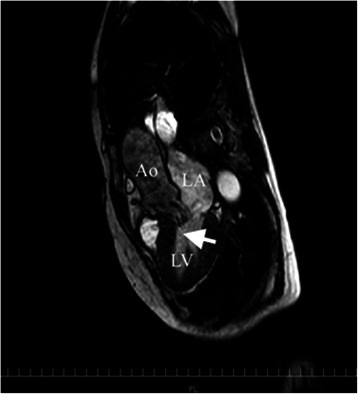
Cardiac MRI obtained 4 days after surgery. It revealed systolic anterior motion (arrow). There were no abnormalities in global shape, size, and systolic function of the left ventricle, except for some protrusion of the basal interventricular septum towards the left ventricular outflow tract. LA = left atrium; LV = left ventricle; Ao = aorta.

Upon arrival at the post-anesthesia care unit, the patient was calm but still had a low SpO_2_ of 88%. Her blood pressure was 160/85 mmHg and her HR was 81 beats per minute. SpO_2_ increased to 92% soon with high-flow oxygen via face mask at a concentration of 60%, but it remained in the range of 90–93% with a smooth respiration at 18 breaths per minutes. Her arterial blood gases were pH 7.354, pCO_2_ 47.8 mmHg, pO_2_ 53.5 mmHg and base excess +0.5 mmol/liter. A portable chest radiograph showed bilateral hilar pulmonary infiltrates consistent with pulmonary edema, a normal cardiac silhouette, and air in the stomach (Figure 1). A nasogastric tube was placed to deflate the distended stomach. Bedside transthoracic echocardiography (TTE) revealed a hypercontractile left ventricle, no right ventricular dilatation, and no segmental wall motion abnormalities; however, slight mosaic flow signals in the left ventricular outflow tract (LVOT) and a posteriorly directed slight jet of mitral regurgitation (MR) were noted on color-flow Doppler mapping (CFD) (Figure 2). Suspecting the presence of SAM, isosorbide dinitrate was sprayed under the patient’s tongue at a dose of 2.5 mg to induce vasodilation. The MR jet in the posterior direction and the mosaic-pattern LVOTO then increased significantly, while blood pressure decreased to 78/42 mmHg (Figure 3) (Additional file 1: Video 1 a, b). We could not measure LVOT velocity, because parallel alignment with the continuous Doppler signal to the blood flow in the LVOT was not possible. Based on a diagnosis of dynamic MR and LVOTO secondary to SAM, we intravenously administered 0.2 mg of phenylephrine twice and initiated rapid infusion of 500 mL of hydroxylethyl starch over the course of an hour and continuous infusion of a short-acting beta blocker. The patient’s blood pressure returned to 152/68 mmHg, and SpO_2_ increased to 97–99% with an oxygen mask at a concentration of 40%. She was cared for overnight in the intensive care unit uneventfully without recurrence of hypoxemia, and transferred to the ward the next day. The bilateral hilar infiltrate had disappeared on the chest radiograph taken the next day. Troponin T and BNP at the post-anesthesia care unit were slightly increased to 0.012 ng/mL (normal <0.014 ng/mL) and 19.0 pg/mL (normal <18.4 pg/mL), respectively, suggesting slight myocardial injury. Cardiac magnetic resonance imaging (MRI) (Figure 4) (Additional 2: Video 2) performed 4 days after surgery, as well as transesophageal echocardiography (TEE), showed chordal SAM with mild MR and LVOTO at rest. The imaging showed some protrusion of the basal septal wall toward the LVOT. The peak pressure gradient through the LVOT was 20 mmHg on TEE. Echocardiographic measurement was performed by TEE to assess the anatomical factors that contributed to SAM (Table 1). The coaptation point of the mitral leaflets was 17 mm, shorter than the threshold value (25 mm), indicating an anteriorly displaced coaptation point, although other measurements did not reach threshold values. The patient is now regularly checked upon, and is taking 5 mg of oral bisoprolol fumarate per day.

**Table 1:** **Echocardiographic predictors of systolic anterior motion (SAM)**

Measurements	Threshold values	Patient values
Basal septal thickness at end-systole	>15 mm	14 mm
Distance from coaptation point to septum at onset of systole	<25 mm	17 mm
Mitral-aortic angle at onset of systole	<120°	130°

Echocardiographic predictors of SAM described in reference 5. Values outside threshold limits indicate increased likelihood of SAM. Measurement was performed on transesoephageal echocardiographic images. Coaptation point of the mitral leaflets of the patient was shorter than the threshold value. Other values were within threshold limits.

Midesophageal aortic valve long-axis view with color Doppler imaging showing aortic insufficiency (arrow). LA = left atrium; LV = left ventricle; Ao = aorta.

## Discussion

SAM typically occurs in association with certain underlying heart diseases, such as HOCM and apical ballooning syndrome or in the perioperative setting of cardiac surgeries, such as mitral valve repair and aortic valve replacement [5]. Recently, its occurrence was described in critical care settings without underlying heart disease [1,6]. Whether it occurs in the presence or absence of underlying heart disease, unexpected severe hypotension is the most typical manifestation and is caused by the dynamic LVOTO secondary to SAM [2,4,6,7]. Our patient suffered from unexplained severe hypoxemia upon emergence from anesthesia, without accompanying severe hypotension.

The use of LMA for laparoscopic cholecystectomy may not be common, but it is well accepted in many centers. Although we have used both Classic and ProSeal LMAs more than ten years safely, the latter is now preferred for ease of gastric tube placement and higher sealing pressure [8]. There is a theoretical possibility of silent aspiration of residual gastric fluid in this case for hypoxemia with a ProSeal LMA. However, transient nature of hypoxemia, uneventful course in the ICU on the following night and complete disappearance of infiltrate in the chest roentgenogram on the next day exclude its possibility. Other causes such as residual neuromuscular blockade, atelectasis or acute pulmonary edema, are also unlikely to have caused severe hypoxemia with hypotension, because of reliable antagonistic effects to rocuronium with sugammadex or a short duration of laparoscopic surgery in an otherwise healthy patient.

Based on findings from the chest radiograph and TTE, we speculate that MR secondary to SAM caused pulmonary edema and hypoxemia in this patient. Because SAM leads to LVOTO and a systolic coaptation defect of the mitral valve, and because the severity varies in a dynamic way depending upon cardiac loading, chronotropy, and contractility [5-7], it is not surprising that severe hypoxemia, rather than hypotension, manifested in this patient.

Cardiac MRI and TEE showed chordal SAM with MR at rest, suggesting that the patient was predisposed to SAM, although there were no apparent structural abnormalities. It has been reported that there are several structural predisposing factors for SAM, such as narrow LVOT, anteriorly located mitral coaptation point, and small mitral–aortic angle of <120° [5]. We identified an anteriorly located mitral coaptation point in this patient’s heart. In addition to the echocardiographic predisposing factors, we speculate that anesthesia-mediated factors, such as anesthesia-mediated vasodilation, increased catecholamine levels due to surgical stimulation, and dehydration due to preoperative fasting, increased the severity of SAM and secondary MR [4].

Treatment for pulmonary edema caused by SAM-induced MR differs from treatment of other forms of cardiogenic pulmonary edema, even though left atrial pressure is increased in both conditions [3]. The accepted treatments for cardiogenic pulmonary edema, such as diuretics, vasodilators, and inotropes, may worsen hypoxemia in patients with pulmonary edema caused by SAM-induced MR or even lead to cardiogenic shock or sudden death from LVOTO, if the cause remains unrecognized. Correct diagnosis of the underlying condition, i.e., SAM, is thus critically important for initiating appropriate treatment. Although the most common clinical finding in SAM or LVOTO is a new onset of systolic murmur peaking late [6], it is often difficult to obtain clear heart sounds during surgery or in the critical care setting, because of interference from noise induced by mechanical ventilation and other surrounding monitoring devices.

Bedside TTE may be an important first-line diagnostic tool for the diagnosis of SAM [6]. It is also very helpful for differentiating SAM-related pulmonary edema from other forms of cardiogenic pulmonary edema. Echocardiographic visualization of the mitral leaflet–ventricular septum contact during systole on 2D or M mode, or shark-tooth velocity contour in the LVOT on continuous Doppler flow are diagnostic for SAM. In addition, mosaic flow pattern in the LVOT and a posteriorly directed MR jet on CFD may suggest LOVOT and MR, respectively, secondary to SAM. In this case, the initial TTE revealed only the mosaic pattern in the LVOT with a slight MR jet. However, careful examination of TTE using sublingual nitrate clearly demonstrated a posteriorly directed MR jet and an increase in the LVOTO mosaic pattern. We could thus establish a diagnosis of SAM and secondary MR as the cause of hypoxemia in this patient.

TEE for the diagnosis of SAM is limited mostly to intraoperative intubated patients, because of its invasiveness. However, TEE could have clearly revealed systolic contact of the anterior leaflet of the mitral valve to the septum [2]. It is thus advisable to use TEE in patients for whom a bedside TTE is not sufficiently informative to diagnose SAM, especially hemodynamically unstable patients.

The present case underscores the fact that to make a diagnosis of SAM, it is important to bear in mind its possibility when unexplained hypoxemia or hypotension occur, even in patients without apparent structural abnormalities of the heart. Clinicians can then listen for systolic murmur or perform bedside TTE under vasodilation with nitrate, if necessary, and eventually perform TEE for timely initiation of appropriate treatment.

## Conclusion

We reported our experience with a patient who had sudden hypoxemia with pulmonary edema at the end of anesthesia following an uneventful laparoscopic surgery. With careful bedside TTE, we were able to explore the underlying cause, i.e., SAM. Clinicians should be aware that SAM may result in unexplained hypoxemia in patients without apparent structural abnormalities of the heart.

## Consent

Written informed consent was obtained from the patient for publication of this Case report and any accompanying images. A copy of the written consent is available for review by the Editor of this journal.

## Additional files

Additional file 1: **Bedside transthoracic echocardiography (color-flow Doppler mapping).** Left parasternal long axis view in the post-anesthesia care unit before (a) and after (b) sublingual nitrate. (a) hypercontractile left ventricle with slight mosaic flow signals in the left ventricular outflow tract and posteriorly directed slight jet of mitral regurgitation (MR) were noted on color-flow Doppler mapping. (b) MR jet in the posterior direction and mosaic pattern left ventricular outflow obstruction increased significantly. Additional file 2: **Cardiac MRI obtained 4 days after surgery revealed chordal systolic anterior motion with mitral regurgitation.** There were no abnormalities in global shape, size, and systolic function of the left ventricle, except for some protrusion of the basal interventricular septum towards the left ventricular outflow tract.

## Abbreviations

Ao: Aorta
CFD: Color-flow Doppler mapping
HOCM: Hypertrophic obstructive cardiomyopathy
HR: Heart rate
LMA: Laryngeal mask airway
LA: Left atrium
LV: Left ventricle
LVOT: Left ventricular outflow tract
LVOTO: Left ventricular outflow tract obstruction
MR: Mitral regurgitation
MRI: Magnetic resonance imaging
PLMA: ProSeal® laryngeal mask airway
SAM: Systolic anterior motion
SpO_2_: oxygen saturation
TEE: Transesophageal echocardiography
TTE: Transthoracic echocardiography
